# Fine-tuning or prompting on LLMs: evaluating knowledge graph construction task

**DOI:** 10.3389/fdata.2025.1505877

**Published:** 2025-06-25

**Authors:** Hussam Ghanem, Christophe Cruz

**Affiliations:** ^1^Université Bourgogne Europe, CNRS, Laboratoire Interdisciplinaire Carnot de Bourgogne ICB UMR 6303, Dijon, France; ^2^DAVI The Humanizers, Puteaux, France

**Keywords:** Text-to-Knowledge Graph, large language models, zero-shot prompting, few-shot prompting, fine-tuning

## Abstract

This paper explores Text-to-Knowledge Graph (T2KG) construction, assessing Zero-Shot Prompting, Few-Shot Prompting, and Fine-Tuning methods with Large Language Models. Through comprehensive experimentation with Llama2, Mistral, and Starling, we highlight the strengths of FT, emphasize dataset size's role, and introduce nuanced evaluation metrics. Promising perspectives include synonym-aware metric refinement, and data augmentation with Large Language Models. The study contributes valuable insights to KG construction methodologies, setting the stage for further advancements.^1^

## 1 Introduction

The term “knowledge graph” has been around since 1972, but its current definition can be traced back to Google in 2012. This was followed by similar announcements from companies such as Airbnb, Amazon, eBay, Facebook, IBM, LinkedIn, Microsoft, and Uber, among others, leading to an increase in the adoption of Knowledge graphs(KGs) by various industries. As a result, academic research in this field has seen a surge in recent years, with an increasing number of scientific publications on KGs (Hogan et al., 2021). These graphs utilize a graph-based data model to manage, integrate, and extract valuable insights effectively from large and diverse datasets (Noy et al., 2019).

KGs serve as repositories for structured knowledge, organized into a collection of triples, denoted as *KG* = (*h, r, t*)⊆*E*×*R*×*E*, where E represents the set of entities, and R represents the set of relations (Hogan et al., 2021). Within a graph, nodes represent various levels, entities, or concepts. These nodes encompass diverse types, including person, book, or city, and are interconnected by relationships such as located in, lives in, or works with. The essence of a KG emerges when it incorporates multiple types of relationships rather than being confined to a single type. The overarching structure of a KG constitutes a network of entities, featuring their semantic types, properties, and interconnections. Thus, constructing a KG necessitates information about entities (along with their types and properties) and the semantic relationships that bind them. For the extraction of entities and relationships, practitioners often turn to NLP tasks like Named Entity Recognition (NER), Coreference Resolution (CR), and Relation Extraction (RE).

KGs are crucial in organizing complex information across diverse domains, such as question answering, recommendations, semantic search, etc. However, the ongoing challenge persists in constructing them, particularly as the primary sources of knowledge are embedded in unstructured textual data such as press articles, emails, and scientific journals. This challenge can be addressed by adopting an information extraction approach, sometimes implemented as a pipeline. It involves taking textual inputs, processing them using Natural Language Processing (NLP) techniques, and leveraging the acquired knowledge to construct or enhance the KG.

If we envision the Text-to-Knowledge Graph (T2KG) construction task as a black box, the input is textual data, and the output is a knowledge graph. In this study, we define the Text-to-Knowledge Graph (T2KG) task as the transformation of unstructured text into a structured set of factual triples. Formally, given an input sentence or paragraph *x*, the task is to produce a set of triples $T={\left\{\left(s_{i},p_{i},o_{i}\right)\right\}}_{i=1}^{n}$, where each triple consists of a subject *s*_*i*_, a predicate *p*_*i*_, and an object *o*_*i*_. Subjects and objects can be named entities or literals, and predicates represent relations between them. A triple is considered valid if it semantically aligns with the information expressed or justifiably implied in the input text. This includes paraphrased expressions and inferred facts, while excluding hallucinated content—i.e., information not supported by the input.

The transformation function can be viewed as *T* = *f*_θ, π_(*x*), where θ represents the language model's parameters and π denotes the prompting strategy employed. In this work, we explore three such strategies: zero-shot prompting (ZSP), where the model is guided by instructions alone; few-shot prompting (FSP), where task examples are included in the prompt; and fine-tuning (FT), where the model is trained on labeled data to perform the extraction directly. This definition provides a consistent framework for evaluating semantic fidelity (see Section 4.2 for evaluation details), generalization, and structure in the generated knowledge graphs.

Achieving this can be approached through methods that directly convert text into a graph or by implementing NLP tasks in two ways (Zhong et al., 2023): (1) through an information extraction pipeline incorporating the mentioned tasks independently, or (2) by adopting an end-to-end approach, also known as joint prediction, using Large Language Models (LLMs) for example. In the realm of LLMs and KGs, their mutual enhancement is evident. LLMs can assist in the construction of KGs. Conversely, KGs can be employed to validate outputs from LLMs or provide explanations for them (Mihindukulasooriya et al., 2023). LLMs can be adapted to the T2KG construction task through various approaches, such as fine-tuning (Ershov, 2023) (FT), zero-shot prompting (Caufield et al., 2023) (ZSP), or few-shot prompting (FSP) (Han et al., 2023) with a limited number of examples. Each of these approaches has their pros and cons with respect to the performance, computation resources, training time, domain adaption and training data required.

In-context learning, as discussed by Min et al. (2022), coupled with prompt design, involves telling a model to execute a new task by presenting it with only a few demonstrations of input-output pairs during inference. Instruction fine-tuning methods, exemplified by InstructGPT (Ouyang et al., 2022) and Reinforcement Learning from Human Feedback (RLHF) (Stiennon et al., 2020), markedly enhance the model's ability to comprehend and follow a diverse range of written instructions. Numerous LLMs have been introduced in the last year, as highlighted by Mihindukulasooriya et al. (2023), particularly within the ChatGPT (OpenAI, 2023) like models, which includes GPT-3 (Brown et al., 2020), LLaMA (Touvron et al., 2023), BLOOM (Workshop et al., 2022), PaLM (Chowdhery et al., 2023), Mistral (Jiang et al., 2023), Starling (Zhu B. et al., 2023), and Zephyr (Tunstall et al., 2023). These models can be readily repurposed for KG construction from text by employing a prompt design that incorporates instructions and contextual information.

**Figure 1:**
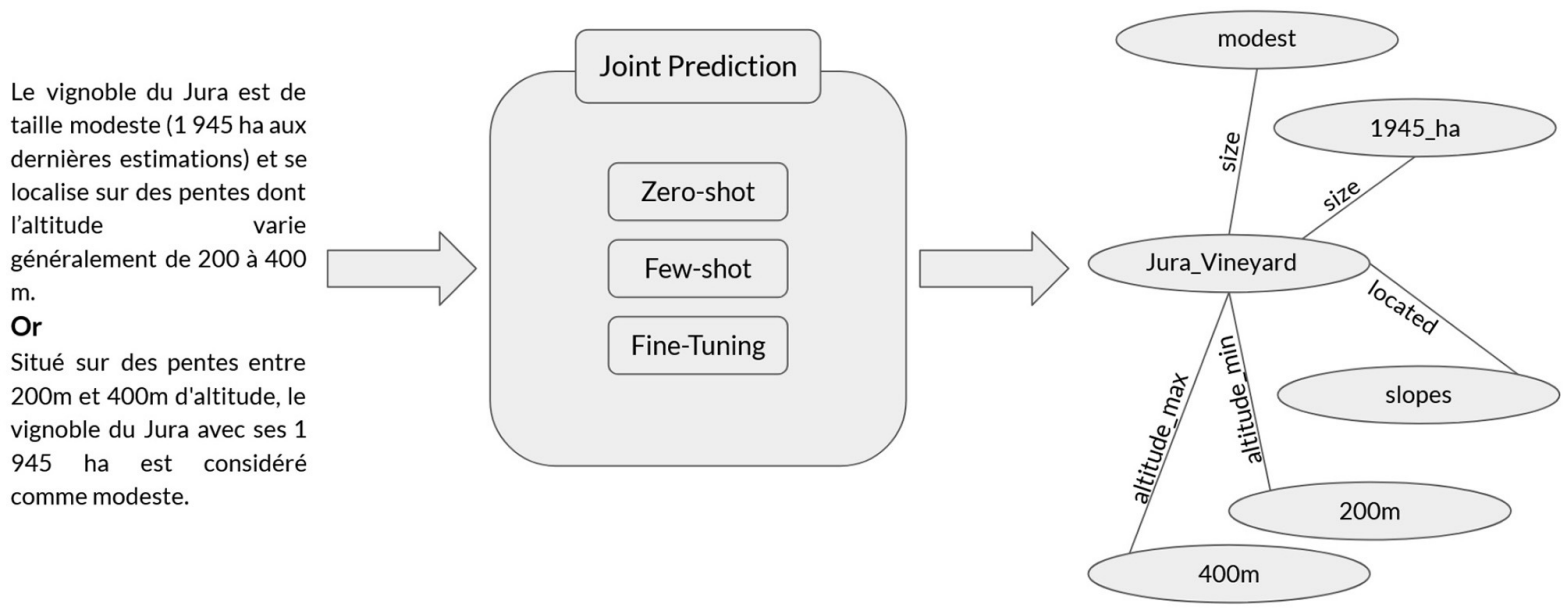
T2KG task.

This study focuses on the formal evaluation of the T2KG task, exploring the developments and challenges associated with KG construction using LLMs. Specifically, we benchmark open-source LLMs across three adaptation strategies–zero-shot, few-shot, and fine-tuning (Figure 1). Unlike prior work that primarily examines proprietary models or single adaptation techniques, our study systematically compares multiple strategies across various models. Additionally, we introduce the GM-GBS metric to assess semantic alignment in generated triples, offering a refined evaluation perspective beyond standard precision-recall measures.

While this paper is primarily positioned as a benchmarking study, our findings also offer empirical insights into the trade-offs between Zero-Shot Prompting, Few-Shot Prompting, and Fine-Tuning. We do not prescribe one universal strategy but provide data-driven guidance to help practitioners choose the most appropriate method based on their task constraints.

The present study is organized as follows, Section 2 presents a comprehensive overview of the current state-of-the-art approaches for T2KG Construction. Section 3 presents the general architecture of our proposed implementation (method), with datasets, metrics, and experiments. Section 4 then encapsulates the findings and discussions, presenting the culmination of results. Finally, Section 5 critically examines the strengths and limitations of these techniques.

## 2 Background

The current state of research on knowledge graph construction using LLMs is discussed. Three main approaches are identified: ZSP, FSP, and FT. Each approach has its own challenges, such as maintaining accuracy without specific training data or ensuring the robustness of models in diverse real-world scenarios. Evaluation metrics used to assess the quality of constructed KGs are also discussed, including semantic consistency and linguistic coherence. This section highlights methods and metrics to construct KGs and evaluate the result.

Figure 1 illustrates the black box joint prediction of the T2KG construction process using LLMs. It demonstrates how two French examples on the left are converted into an expected result (Knowledge Graph) on the right using ZSP, FSP or FT approaches with LLMs.

### 2.1 Zero shot

Zero Shot methods enable KG construction without task-specific training data, leveraging the inherent capabilities of large language models. Carta et al. (2023) introduces an innovative approach using LLMs for knowledge graph construction, employing iterative ZSP for scalable and flexible KG construction. Zhu Y. et al. (2024) evaluate the performance of LLMs, specifically GPT-4 and ChatGPT, in KG construction and reasoning tasks, introducing the Virtual Knowledge Extraction task and the VINE dataset, but they do not take into account open sourced LLMs as LLaMA (Touvron et al., 2023). Li et al. (2023) assess ChatGPT's abilities in information extraction tasks, identifying overconfidence as an issue and releasing annotated datasets. Wei et al. (2023) tackle zero-shot information extraction using ChatGPT, achieving impressive results in entity relation triple extraction. Laurenzi et al. (2024) investigate the use of LLMs for Enterprise KG construction, proposing a six-step process that integrates LLMs to reduce manual effort and lower the expertise barrier. Jarnac et al. (2023) propose a method for Knowledge Graph Construction (KGC) using an analogy-based approach, demonstrating superior performance on Wikidata. Bi et al. (2024) address the limitations of existing generative knowledge graph construction methods by leveraging large generative language models trained on structured data. Most of these approaches having the same limitation, which is the use of closed and huge LLMs as ChatGPT or GPT4 for this task. Challenges in this area include maintaining accuracy without specific training data and addressing nuanced relationships between entities in untrained domains.

### 2.2 Few shot

Few Shot methods focus on constructing KGs with limited training examples, aiming to achieve accurate knowledge representation with minimal data. Han et al. (2023) introduce PiVe, a framework enhancing the graph-based generative capabilities of LLMs, and the authors create a verifier which is responsable to verify the results of LLMs with multi-iteration type. Yao et al. (2023) explore the potential of LLMs for knowledge graph completion, treating triples as text sequences and utilizing LLM responses for predictions. Khorashadizadeh et al. (2023) automate the process of generating structured knowledge graphs from natural language text using foundation models. Deng et al. (2023) present OpenBG, an open business knowledge graph derived from Alibaba Group, containing 2.6 billion triples with over 88 million entities. Trajanoska et al. (2023) explore the integration of LLMs with semantic technologies for reasoning and inference. Chen et al. (2023) investigate LLMs' application in relation labeling for e-commerce Knowledge Graphs (KGs). Kommineni et al. (2024) explore the semi-automated construction of knowledge graphs using open-source LLMs. Meyer et al. (2023) investigated the integration of ChatGPT for KG engineering, demonstrating its ability to automate T2KG construction. Other studies, such as Hu et al. (2024), have explored LLM applications for domain-specific KG construction, particularly in cybersecurity contexts. As ZSP approaches, FSP approaches use closed and huge LLMs as ChatGPT or GPT4 (OpenAI, 2023) for this task. Challenges in this area include achieving high accuracy with minimal training data and ensuring the robustness of models in diverse real-world scenarios.

### 2.3 Fine-tuning

Fine-Tuning methods involve adapting pre-trained language models to specific knowledge domains, enhancing their capabilities for constructing KGs tailored to particular contexts. Ershov (2023) present a case study automating KG construction for compliance using BERT-based models. This study emphasizes the importance of machine learning models in interpreting rules for compliance automation. Harnoune et al. (2021) propose an approach for knowledge extraction and analysis from biomedical clinical notes, utilizing the BERT model and a Conditional Random Field layer, showcasing the effectiveness of leveraging BERT models for structured biomedical knowledge graphs. Yang et al. (2023) propose Knowledge Graph-Enhanced Large Language Models (KGLLMs), enhancing LLMs with KGs for improved factual reasoning capabilities. These approaches that applied FT, they do not use new generations of LLMs, specially, decoder only LLMs as Llama, and Mistral. Challenges in this domain include ensuring the scalability, interpretability, and robustness of fine-tuned models across diverse knowledge domains.

### 2.4 Evaluation metrics

As we employ LLMs to construct KGs, and given that LLMs function as Natural Language Generation (NLG) models, it becomes imperative to discuss NLG criteria. In NLG, two criteria (Ferreira et al., 2019) are used to assess the quality of the produced answers (triples in our context).

The first criterion is semantic consistency or Semantic Fidelity which quantifies the fidelity of the data produced against the input data. The most common indicators are:

- **Hallucination**: It is manifested by the presence of information (facts) in the generated text that is absent in the input data. In our scenario, hallucination metric counts triples present in the generated triples (GT) but missing in the ground truth triples (ET);
- **Omission**: It is manifested by the omission of one of the pieces of information (facts) in the generated text. In our case, omission counts triples present in ET but missing in GT;
- **Redundancy**: This is manifested by the repetition of information in the generated text. In our case, the redundancy exists if a triple appears more than once in GT;
- **Accuracy**: The lack of accuracy is manifested by the modification of information such as the inversion of the subject and the direct object complement in the generated text. Accuracy increases if there is an exact match between ET and GT. ;
- **Ordering**: It occurs when the sequence of information is different from the input data. In our case, the ordering of GT is not considered.

The second criterion is linguistic coherence or Output Fluency to evaluate the fluidity of the text and the linguistic constructions of the generated text, the segmentation of the text into different sentences, the use of anaphoric pronouns to reference entities and to have linguistically correct sentences. However, in our evaluation, we do not take into account the second criterion.

In their experiments, Mihindukulasooriya et al. (2023) calculated three hallucination metrics—subject hallucination, relation hallucination, and object hallucination—using certain preprocessing steps such as stemming. They used the ground truth ontology alongside the ground truth test sentence to determine if an entity or relation is present in the text. However, a limitation could arise when there is a disparity in entities or relations between the ground truth ontology and the ground truth test sentence. If the generated triples contain entities or relations not present in the ground truth text, even if they exist in the ground truth ontology, it will be considered a hallucination.

The authors of Han et al. (2023) evaluate their experiments using several evaluation metrics, including Triple Match F1 (T-F1), Graph Match F1 (G-F1), G-BERTScore (G-BS) from Saha et al. (2021) which extends BertScore (Zhang T. et al., 2019) for graph matching, and Graph Edit Distance (GED) from Abu-Aisheh et al. (2015). The GED metric measures the distance between the predicted graph and the ground-truth graph, which is equivalent to computing the number of edit operations (addition, deletion, or replacement of nodes and edges) needed to transform the predicted graph into a graph that is identical to the ground-truth graph, but it does not provide a specific path for these operations to calculate the exact number of operations. To adhere with the semantic consistency criterion, we use the terms “omission” and “hallucination” in place of “addition” and “deletion,” respectively.

### 2.5 Limitations of triple-based representations

While the (subject, predicate, object) format is widely used in KG construction, it is inherently limited in expressing complex semantic phenomena such as temporal relations, modality, causality, and uncertainty. For instance, a sentence like “Nie Haisheng is believed to have trained as a fighter pilot before becoming an astronaut” encodes temporal sequencing and epistemic modality that flat triples fail to capture. Prior works have highlighted that RDF-style triples oversimplify nuanced linguistic structures and are insufficient for representing dynamic or contextual knowledge (Ji et al., 2022; Allen et al., 2022). These limitations have motivated research into more expressive representations, such as event-centric knowledge graphs (Rospocher et al., 2016) and frame-based or graph-structured semantic parsers (Banarescu et al., 2013). In this study, we treat T2KG as a practical approximation and recognize that supporting richer knowledge formalisms remains an important direction for future research.

## 3 Propositions

This section describes our approach to evaluate the quality of generated KGs. We explain how we use evaluation metrics such as T-F1, G-F1, G-BS, GED, Bleu-F1 (Papineni et al., 2002), and ROUGE-F1 (Lin, 2004) to assess the quality of the generated KGs in comparison to ground-truth KGs. Additionally, we discuss the use of Optimal Edit Paths (OEP) metric^2^ to determine the precise number of operations required to transform the predicted graph into an identical representation of the ground-truth graph. This metric serves as a basis for calculating omissions and hallucinations in the generated graphs. We employ examples from the WebNLG+2020 (Gardent et al., 2017) training dataset for testing with FSP techniques. Additionally, we utilize the training dataset of WebNLG+2020 to train LLMs using the FT technique. For further experimentation, we employ examples from KELM-sub training dataset (Han et al., 2023). Subsequent subsections delve into a detailed discussion of each phase.

### 3.1 Overall experimentation process

We leverage the WebNLG+2020 dataset, specifically the version curated by Han et al. (2023). Their preparation of graphs in lists of triples proves beneficial for evaluation purposes. We utilize these lists and employ NetworkX (Hagberg et al., 2008) to transform them back into graphs, facilitating evaluations on the resultant graphs. This step is instrumental in performing ZSP, FSP, and FT LLMs on this dataset.

**Figure 2:**
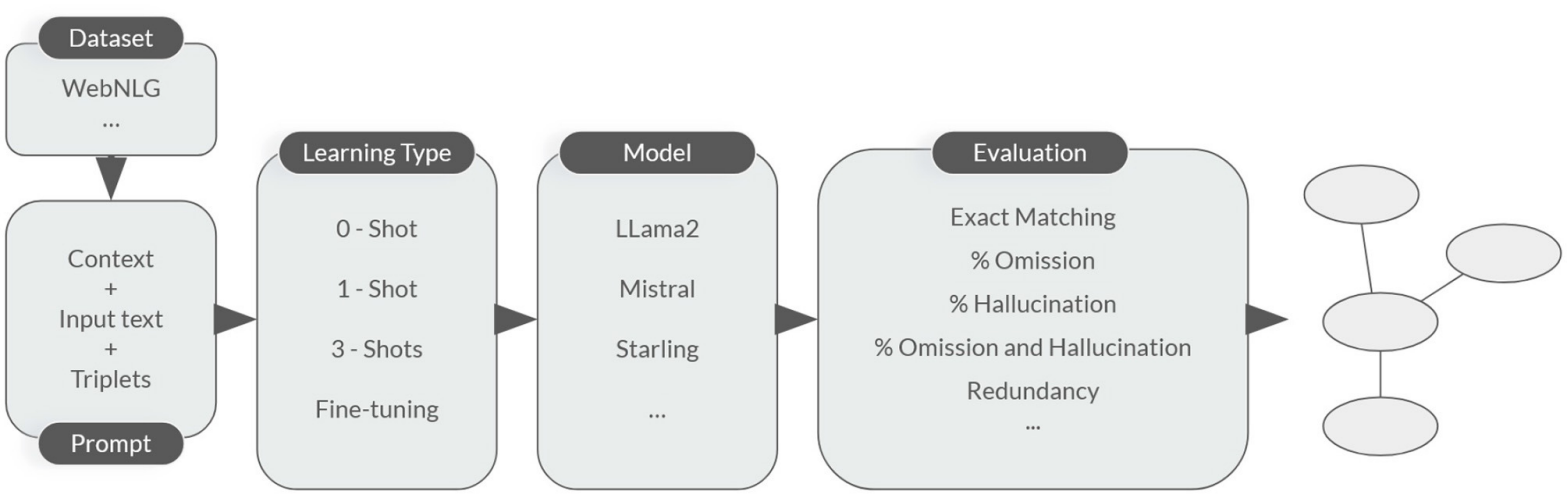
Overall experimentation's process.

Figure 2 illustrates the different stages of our experimentation process, including data preparation, model selection, training, validation, and evaluation. The process begins with data preparation, where the WEBNLG dataset is preprocessed and split into training, validation, and test sets. Next, the learning type is selected, and different models are trained using the training set. The trained models are then evaluated on the validation set to evaluate their performance. Finally, the best-performing model is selected and validated on the test set to estimate its generalization ability.

### 3.2 Preprocessing

In this phase, we directly utilize the WebNLG and KELM-sub datasets as provided by Han et al., without additional modifications. These datasets have been carefully curated by experts to align with the T2KG task, ensuring high-quality, manually verified triples for knowledge graph construction. Given their structured nature, no further preprocessing was required before feeding them into our pipeline. However, while these datasets provide a strong foundation, future work could explore entity harmonization techniques to further refine the extracted triples. By integrating entity linking strategies (Dredze et al., 2010) or LLM-assisted canonicalization methods (Zhang and Soh, 2024), we could improve coherence and reduce redundancy in the constructed knowledge graphs.

### 3.3 Prompting learning

**Figure 3:**
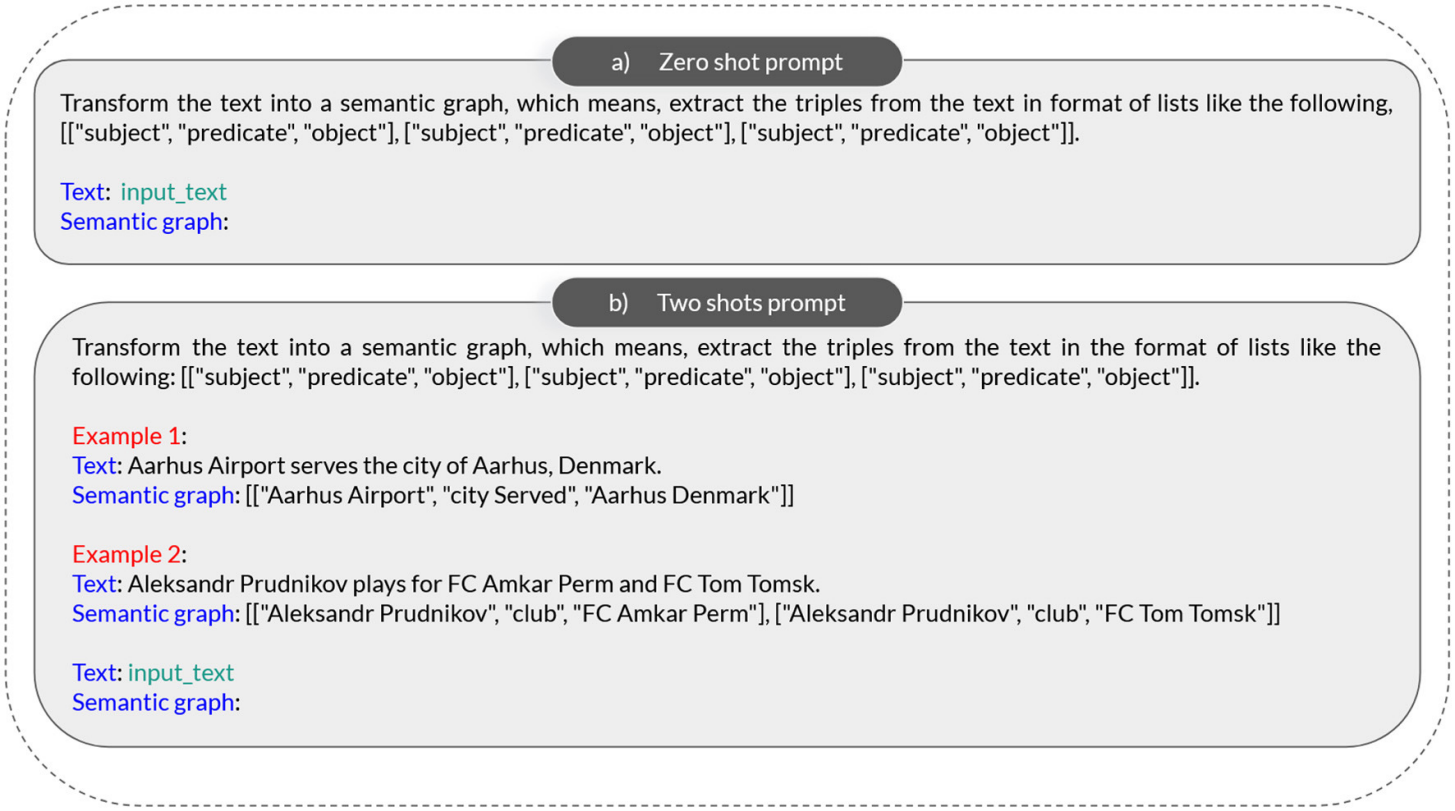
Prompting examples. **(a)** Zero shot prompt. **(b)** Two shots prompt.

During this phase, we employ the ZSP and FSP techniques on LLMs to evaluate their proficiency in extracting triples (e.g., construction of the KG). The application of these techniques involves merging examples from the training dataset of WebNLG+2020 with our adapted prompt. Our prompt is strategically modified to provide contextual guidance to the LLMs, facilitating the effective extraction of triples, without the inclusion of a support ontology description, as demonstrated in Mihindukulasooriya et al. (2023). The specific prompts used for ZSP and FSP are illustrated in Figures 3a, b.

In our approach for ZSP, we began with the methodology outlined in Han et al. (2023), initiating our prompt with the directive “Transform the text into a semantic graph.” However, we enhanced this prompt by incorporating additional sentences tailored for our LLMs, as illustrated in Figure 3a.

The effectiveness FSP depends heavily on example selection (Yoshida, 2024). In our experiments, we selected examples based on criteria shown in the following paragraph. However, prior research suggests that structured selection methods–such as clustering diverse examples or prioritizing domain-specific samples–could significantly improve performance. Exploring optimal example selection strategies remains a valuable direction for future work.

For FSP, we executed 7-shots learning. The rationale behind employing 7-shots learning lies in the fact that the maximum KG size in WebNLG+2020 is 7 triples. Consequently, we fed our prompt with 7 examples of varying sizes; example 1 with size 1, example 2 with size 2, example 3 with size 3, and so forth. In Figure 3b, we depict a prompt containing two examples.

To demonstrate the efficacy of our refined prompt (including additional sentences), we conducted zero-shot experiments on ChatGPT (OpenAI, 2023), comparing the outcomes with those of Han et al. (2023). Our results consistently reveal that our prompt yields more coherent answers in terms of structure.

For further experimentation, we use examples from KELM-sub training dataset, especially when we discuss the generalizability of the original and finetuned vesions of our the most performing model. To do so, we used 6-shot learning, corresponding to the maximum KG size in KELM-sub.

As mentioned before, FSP test cases in our experiments utilized manually selected examples from the WebNLG+2020 dataset to maintain consistency. However, advanced techniques such as LLM-generated prompts or retrieval-augmented example selection could further refine few-shot effectiveness. Future work will explore dynamic example selection strategies to optimize model performance across diverse scenarios.

In addition to standard zero-shot and few-shot prompting, we acknowledge the growing significance of Chain-of-Thought (CoT) prompting (Wei et al., 2022). CoT enables LLMs to decompose complex reasoning tasks into intermediate steps, which has been shown to enhance structured information extraction. While our study primarily focuses on direct triple extraction using standard prompting approaches, future work should investigate how CoT influences knowledge graph generation, particularly in reducing hallucinations and improving entity-relation consistency.

### 3.4 Finetuning

If the initial results from the ZSP and FSP on LLMs prove reasonable, we proceed to the FT phase. This phase aims to provide the LLMs with a more specific context and knowledge related to the task of extracting triples within the domains covered by the WebNLG+2020 dataset. Using the example “(a)” illustrated in Figure 3, we pass in the FT prompt, at once for each line of the training dataset, the input text and the corresponding KG (the list of triples). To do this phase (FT), we employ QLoRA (Dettmers et al., 2024), a methodology that integrates quantization (Zhang X. et al., 2019) and Low-Rank Adapters (LoRA) (Hu et al., 2021). The LLM is loaded with 4-bit precision using bitsandbytes (Dettmers et al., 2023), and the training process incorporates LoRA through the PEFT library (Parameter-Efficient Fine-Tuning) (Mangrulkar et al., 2022) provided by Hugging Face.

### 3.5 Postprocessing

Given our focus on KG construction, our evaluation process involves assessing the generated KGs against ground-truth KGs. To facilitate this evaluation, we take a cleaning process for the LLMs output. This involves transforming the graphs generated by LLMs into organized lists of triples, subsequently transferred to textual documents.

The transformation is executed through rule-based processing. This step is applied to remove corrupted text (outside the lists of triples) from the whole text generated by LLMs in the preceding step. The output is then presented in a list of lists of triples format, optimizing our evaluation process. This approach proves especially effective when calculating metrics such as G-F1, GED, and OEP, as we will see in more detail in Section 3.6.

**Figure 4:**
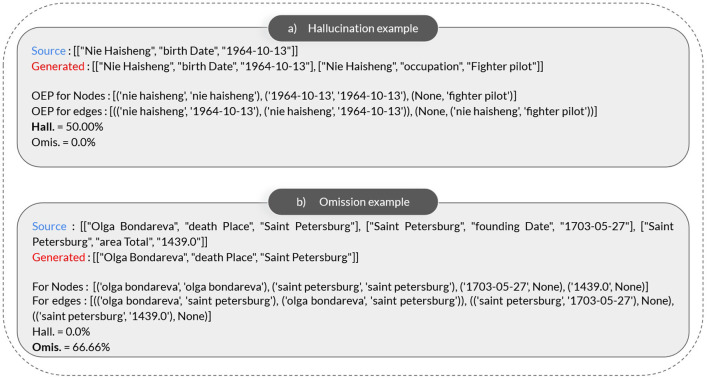
Examples and results. **(a)** Hallucination example. **(b)** Omission example.

A potential problem arises when instructing LLMs to produce lists of triples (KGs), as there may be instances where the generated text lacks the desired structure. In such cases, we address this issue by substituting the generated text with an empty list of triples, represented as ‘[[“”, “”, “”]],' allowing us to effectively evaluate omissions. However, this approach tends to underestimate hallucinations compared to the actual occurrences. To address this issue, we calculate the exact hallucination and omission for each generated graph through qualitative evaluation of two randomly generated graphs (Figure 4).

### 3.6 Experiment's evaluation

For assessing the quality of the generated graphs in comparison to ground-truth graphs, we adopt evaluation metrics as employed in Han et al. (2023). These metrics encompass T-F1, G-F1, G-BS (Saha et al., 2021), and GED (Abu-Aisheh et al., 2015). Additionally, we incorporate the Optimal Edit Paths (OEP) metric, a tool aiding in the calculation of omissions and hallucinations within the generated graphs.

In the following, we summarize some definitions of the main evaluation metrics :

- **G-F1 and T-F1**: Assess graph- and triple-level precision, recall, and F1-score.
- **GED (Graph Edit Distance)**: Measures the minimal number of edits needed to transform the generated graph into the reference graph.
- **GM-GBS (Graph Matching BERTScore)**: Evaluates semantic similarity between generated and ground truth triples.
- **Hallucination and Omission Analysis**: Identifies cases where the model generates incorrect triples (hallucinations) or fails to generate expected ones (omissions).

A detailed breakdown of the metric calculations is provided in Supplementary Data Sheet.

Our evaluation procedure aligns with the methodology outlined in Han et al. (2023), particularly in the computation of GED and G-F1. This involves constructing directed graphs from lists of triples, referred to as linearized graphs, utilizing NetworkX (Hagberg et al., 2008).

In contrast to Mihindukulasooriya et al. (2023), our methodology diverges by not relying on the ground truth test sentence of an ontology. As previously mentioned, we opt for a distinct approach wherein we assess omissions and hallucinations in the generated graphs using the OEP metric. Unlike the global edit distance provided by GED, OEP gives the precise path of the edit, enabling the exact quantification of omissions and hallucinations, either in absolute terms or as a percentage across the entire test dataset.

For example, in the illustrated nodes path labeled “a)” in Figure 4b, we observe 2 omissions, while the nodes path in Figure 4a exhibits 1 hallucination. In our evaluation, the criterion for incrementing the global hallucination metric for all graphs is set at finding >=1 hallucinations or >=1 omission in a generated graph. This approach ensures a comprehensive assessment of the presence of omissions and hallucinations across the entirety of the generated graphs. We calculate also the exact percentage of hallucination or omission in a generated graph, experimenting on 2 random examples from the WebNLG+2020 test dataset (Figure 4).

As mentioned earlier, the evaluation of the three methods is conducted using examples sourced from the test dataset of WebNLG+2020 and the test dataset of KELM-sub for further experimentation. The primary goal is to enhance G-F1, T-F1, G-BS, Bleu-F1, and ROUGE-F1 metrics, while reducing GED, Hallucination, and Omission.

## 4 Experiments and discussion

This section provides insights into the LLMs utilized in our study for ZSP, FSP, or FT, followed by the presentation of our experimental results.

A detailed experimental setup is provided in Supplementary Data Sheet. While these settings provided meaningful improvements over prompting strategies, further experimentation with hyperparameter optimization—such as adjusting LoRA scaling factors, layer-specific tuning, and learning rate schedules—could yield deeper insights into generalization performance.

### 4.1 Selection criteria of the used LLMs

In this section, we provide a brief overview of the LLMs utilized in our experiments. Our selection criteria focused on employing small, open-source, and easily accessible LLMs. All models were sourced from the HuggingFace platform.^3^

- **Llama 2** (Touvron et al., 2023) is a collection of pretrained and fine-tuned generative text models ranging in scale from 7 billion to 70 billion parameters. In our experiments, we deploy the 7B and 13B pretrained models, which have been converted to the Hugging Face Transformers format.
- Introduced by Jiang et al. (2023), **Mistral-7B-v0.1** is a pretrained generative text model featuring 7 billion parameters. Notably, Mistral-7B-v0.1 exhibits superior performance to Llama 2 13B across all benchmark tests in their experiments.
- In the work presented by Zhu B. et al. (2023), **Starling-7B** is introduced as an open LLM trained through Reinforcement Learning from AI Feedback (RLAIF). This model leverages the GPT-4 labeled ranking dataset, berkeley-nest/Nectar, and employs a novel reward training and policy tuning pipeline.

### 4.2 Metrics analysis and impact of Fine-Tuning on WebNLG+2020

In our review of the state-of-the-art, we observed that, apart from Mihindukulasooriya et al. (2023), which incorporates hallucination evaluation in their experiments, other studies primarily focus on metrics such as precision, recall, F1 score, triple matching, or graph matching. In our approach to evaluating experiments, we consider also hallucination and omission through a linguistic lens.

Upon examining Table 1, we observe the superior performance of the FT method compared to ZSP and FSP for the T2KG construction task. Of particular interest is the finding that, with the exception of Llama2-7b, applying ZSP to the fine-tuned Llama2-7b results in worse performance than FSP on the original Llama2-7b. Overall, this table provides a clear visualization of the relative performance of each method, highlighting the strengths and limitations of each approach for T2KG construction.

**Table 1:** Comparison of performance metrics and models on WebNLG+2020. Lower values indicate better performance for GED, Hall., and Omis.

**Model | Metric**	**G-F1**	**T-F1**	**G-BS**	**GED**	**F1-Bleu**	**F1-Rouge**	**Hall**.	**Omis**.
PiVE	14.00	18.57	89.82	11.22	-	-	-	-
Mistral-0	2.30	0.00	77.87	15.93	54.97	55.15	20.63	31.48
Mistral-7	18.72	28.44	87.54	10.13	55.09	63.94	17.88	21.14
Mistral-FT-0	31.93	44.08	86.89	8.25	63.88	69.08	13.55	18.27
Mistral-FT-7	34.68	**49.11**	**91.99**	6.69	**71.78**	**77.43**	15.01	**14.45**
Starling-0	5.23	7.83	86.29	13.35	34.64	14.61	17.48	33.24
Starling-7	21.30	33.77	90.41	8.96	60.47	69.34	17.31	14.61
Starling-FT-0	21.47	28.29	72.86	11.87	44.07	47.69	10.17	42.78
Starling-FT-7	**35.69**	48.49	91.95	**6.60**	71.51	76.67	11.35	18.27
Llama2-7b-0	0.00	0.46	54.20	18.29	20.23	17.98	4.83	81.53
Llama2-7b-7	11.80	20.88	82.78	12.66	45.48	54.29	20.74	30.02
Llama2-7b-FT-0	3.82	15.41	59.19	15.78	16.82	17.95	6.07	79.20
Llama2-7b-FT-7	18.77	32.63	87.19	10.16	58.48	66.35	25.24	18.66
Llama2-13b-0	0.00	0.79	57.42	17.79	20.50	18.23	**4.78**	81.23
Llama2-13b-7	13.49	23.99	84.89	11.59	50.18	58.71	26.36	19.06
Llama2-13b-FT-0	20.52	32.18	75.88	11.38	46.53	50.78	11.64	39.63
Llama2-13b-FT-7	23.55	37.29	88.77	8.94	63.26	70.12	23.55	16.19

Bold values indicate the best-performing results for each corresponding metric.

Furthermore, it is evident that better results are achieved by providing more examples (more shots) to the same model, whether original or fine-tuned. The results underscore the positive correlation between the quantity of examples and the model's performance. Comparing the fine-tuned Mistral and fine-tuned Starling, they exhibit similar performance when given 7 shots, surpassing the two Llama2 models by a significant margin. The standout performer with ZSP on the fine-tuned LLM is Mistral, showcasing a considerable lead over other LLMs, including Starling. To corroborate these findings, future versions of our study plan to assess our fine-tuned models using an alternative dataset with diverse domains.

As depicted in Table 1, Hall. represents Hallucinations, while Omis. denotes Omissions.

Taking into account our strategy of introducing an empty graph when LLMs fail to produce triples, we note that even with LLama2-13b with ZSP exhibiting the least favorable results across all metrics, it displays minimal hallucinations. Nonetheless, it's crucial to recognize that the model with the fewest hallucinations may not necessarily be the most suitable choice. To overcome this limitation in our evaluation metric, we aim to improve it by considering the prevalence of empty graphs in the generated results before assessing them against ground truth graphs.

The G-BS consistently remains high, indicating that LLMs frequently generate text with words (entities or relations) very similar to those in the ground truth graphs. Among the models, the finetuned Starling with 7 shots achieves the highest G-F1, which focuses on the entirety of the graph and evaluates how many graphs are exactly produced the same, suggesting that it accurately generates approximately 36% of graphs identical to the ground truth. For various metrics, the finetuned Mistral with 7 shots performs exceptionally well, particularly in T-F1, where F1 scores are computed for all test samples and averaged for the final Triple Match F1 score. Additionally, it excels in metrics such as “Omis.,” F1-Bleu, and F1-Rouge. F1-Bleu and F1-Rouge represent n-gram-based metrics encompassing precision (Bleu), recall (Rouge), and F-score (Bleu and Rouge). These metric could potentially yield even better results if synonyms of entities or relations are considered as exact matches.

The authors in Han et al. (2023) conduct evaluations using WebNLG+2020. Consequently, we adopt their approach (PiVE) as a baseline for comparison with our experiments. Upon analyzing the results, it becomes evident that nearly all fine-tuned LLMs outperform PiVE, which is applied on both ChatGPT and GPT-4 as mentioned before.

As in Tables 1, 2 shows that the fine-tuned Mistral with 7 shots from WebNLG+2020 (Mistral-FT-7) performs better than other models in almost all metrics except GM-GBS, where the finetuned Mistral with 6 examples from KELM-sub (Mistral-FT-6) outperforms Mistral-FT-7 and all other models. One reason to use it for the GM-GBS metric in these experiments is that—as mentioned above—G-BS consistently remains high. We observe also that even when we gave Mistral examples from KELM-sub training dataset, it works better than zero-shot for the test dataset of WebNLG.

**Table 2:** Comparison of performance metrics and models on WebNLG test dataset. Lower values indicate better performance for GED, Hall., and Omis.

**Model | Metric**	**G-F1**	**T-F1**	**G-BS**	**GED**	**Hall**.	**Omis**.	**GM-GBS**
Mistral-0	2.30	3.27	77.87	15.84	20.35	31.31	33.27
Mistral-7	18.72	28.44	87.54	10.13	17.88	21.14	51.88
Mistral-FT-0	31.93	44.08	86.89	8.25	**13.55**	18.27	54.97
Mistral-FT-7	**34.68**	**49.11**	**91.99**	**6.69**	14.90	14.39	57.72
Mistral-6 (KELM-sub)	7.59	12.45	81.23	16.29	61.16	**7.64**	26.86
Mistral-FT-6 (KELM-sub)	31.37	47.49	91.27	7.51	27.37	8.26	**58.40**

Bold values indicate the best-performing results for each corresponding metric.

As mentioned before, to corroborate these findings, we assess our fine-tuned models using KELM-sub test dataset for few-shot.

### 4.3 Generalization across domains

In Table 3, we present the evaluation results of original LLMs with 7 shots and fine-tuned LLMs with zero-shot and 7 shots on the KELM-sub test dataset which is prepared by Han et al. (2023), building upon (Agarwal et al., 2020). It's crucial to note that the experiments utilized the same prompts as previously described. The 7-shot experiments sourced examples from the WebNLG+2020 training dataset. These new experiments aim to assess the generalization ability of original LLMs with 7 shots and fine-tuned LLMs with zero-shot and 7 shots across diverse domains in the T2KG construction task.

**Table 3:** Results on KELM-sub.

**Model | Metric**	**G-F1**	**T-F1**	**G-BS**	**GED**	**Hall**.	**Omis**.	**GM-GBS**
Mistral-7	5.50	11.35	81.77	13.74	6.72	61.09	28.66
Mistral-FT-0	2.17	8.55	78.29	14.35	7.22	56.28	12.88
Mistral-FT-7	2.89	9.92	78.42	13.63	**6.22**	61.00	13.66
Mistral-6 (KELM-sub)	**12.00**	**31.08**	**85.49**	**10.82**	25.50	**32.44**	**38.88**
Mistral-FT-6 (KELM-sub)	4.00	17.66	84.30	12.50	11.06	4817	36.22

Bold values indicate the best-performing results for each corresponding metric.

Providing LLMs with insights into certain relation types. Han et al. (2023) use examples from KELM-sub training dataset in their model PiVE, which leeded us to do another experiment which was conducted using 6 random examples from the KELM-sub training dataset. We applied the prompt with these examples to both the original Mistral (Mistral-6) and our finetuned Mistral (Mistral-FT-6) models. As expected, Mistral-6 outperformed Mistral-7 because the examples were from the KELM-sub training dataset used in Mistral-6. However, it was interesting to observe that Mistral-FT-6 performed less effectively than Mistral-6 with the same examples. This suggests that finetuning on WebNLG domains reduces the generalizability of the LLMs.

**Figure 5:**
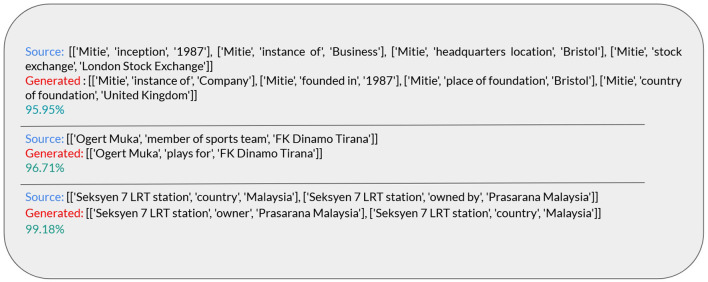
Examples of the calculated GM-GBS.

The results in Table 3 indicate that the fine-tuned Mistral models perform less effectively than the original Mistral with 7 shots from WebNLG+2020 and with 6 shots from KELM-sub. Additionally, all fine-tuned versions of Mistral (Mistral-FT-7, Mistral-FT-0, and Mistral-FT-6) show inferior results on KELM-sub compared to WebNLG+2020. This disparity can be attributed to the presence of different relation types, with some types expressed differently in KELM-sub. To address this, we utilize G-BS to calculate the similarity between two graphs and consider them as synonyms if they are sufficiently similar (>95% of similarity). This metric, called GM-GBS, is the last metric presented in Table 3. GM-GBS indicates a higher value of graph matching. To assess the reliability of this metric, we conducted a qualitative evaluation as illustrated in Figure 5.

Overall, using examples from KELM-sub shows that the results are relatively similar. This indicates that fine-tuning negatively affects the generalization capability of the models.

### 4.4 Implications and future directions

All results highlight the effectiveness of fine-tuning, particularly in improving knowledge graph extraction accuracy. However, the study is limited to the QLoRA approach, and a broader exploration of fine-tuning strategies could provide deeper insights. For example, full fine-tuning might yield different generalization trade-offs, while selective fine-tuning on entity-rich layers could further optimize performance. Future studies should systematically compare different fine-tuning paradigms to assess their impact on both accuracy and generalization. At the same time, our results indicate that increasing the number of few-shot examples leads to improved performance, but the selection strategy plays a crucial role. Automated prompt generation or retrieval-based few-shot prompting could potentially enhance consistency and reduce variance in model output. We recommend future research to investigate optimal prompt selection methodologies to refine the efficacy of few-shot learning in knowledge graph extraction.

### 4.5 Qualitative results

As illustrated in Figure 4, our metric precisely calculates the percentage of hallucinations and omissions in the generated graphs at the triple level. For example, if a generated graph contains 2 triples and 1 of them is not present in the ground truth graph, the hallucination rate is approximately 50%. Similarly, for omissions, if the generated graph is missing some triples present in the ground truth graph, the omission rate is calculated accordingly.

As mentioned above, we use G-BS to calculate the similarity between generated and ground-truth graphs. If the similarity value exceeds 95%, we consider it an exact match, based on the notion that entities or relations in the generated graph are very close to those in the ground-truth graph, or what we refer to as synonyms. In Figure 5, we present examples with varying levels of similarity, including one with approximately 95% similarity, to demonstrate that even with 95% similarity, the two graphs convey the same or very similar meanings.

While our study provides valuable insights into the effectiveness of different LLM-based approaches for T2KG construction, it is limited by the absence of a comprehensive error analysis. A systematic investigation into common failure cases—such as hallucinated triples, missing relations, and entity extraction errors—could offer deeper insights into model weaknesses. However, due to computational resource constraints, we were unable to conduct this analysis in the current study. Future work will focus on addressing this limitation by incorporating an error taxonomy and analyzing failure patterns across different LLMs once adequate resources become available.

### 4.6 Guidelines for strategy selection

Our experiments suggest several heuristics for choosing among ZSP, FSP, FT in the context of T2KG tasks. ZSP is most appropriate when rapid prototyping is needed, no labeled data is available, and the task domain is general or closely aligned with the language model's pre-training. FSP becomes advantageous when a small set of labeled examples can be curated, especially for reinforcing task-specific patterns without engaging in full model retraining; it performs well in domains that deviate moderately from general language. In contrast, FT is preferable when a sufficient volume of labeled data exists, the target domain is highly specialized or technical, and computational resources are available to support the retraining process. While these guidelines are not exhaustive, they are grounded in both our empirical findings and prior research, and they aim to support practitioners in selecting the most appropriate strategy for adapting LLMs to the T2KG task.

## 5 Conclusion and perspectives

This study delves into the T2KG construction task, exploring the efficacy of three distinct approaches: ZSP, FSP, and FT of LLMs. Our comprehensive experimentation, employing models such as Llama2, Mistral, and Starling, sheds light on the strengths and limitations of each approach. The results demonstrate the remarkable performance of the FT method, particularly when compared to ZSP and FSP across various models. Notably, the fine-tuned Llama2-7b with ZSP gave worse results than FSP with the original Llama2. Additionally, the positive correlation between the quantity of examples and model performance underscores the significance of dataset size in training. An essential part of our study involves the evaluation metrics employed to assess the generated graphs. Our analysis incorporated a comprehensive set of metrics, including G-F1, T-F1, G-BS, GED, along with measures for hallucinations and omissions.

Despite these improvements, we observed that fine-tuning on domain-specific data, such as WebNLG, can negatively impact the model's generalization capabilities. This was evident from the comparative performance of the fine-tuned models on the KELM-sub dataset, where the original Mistral model with 7 shots from WebNLG+2020 outperformed the fine-tuned variants. This finding highlights the importance of balancing domain-specific fine-tuning with maintaining broad generalization.

The inclusion of the GM-GBS metric provided valuable insights into the semantic similarity between generated and ground truth graphs. Our qualitative analysis of hallucinations and omissions further enhanced our understanding of model performance at the triple level.

One is to involve refining evaluation metrics to accommodate synonyms of entities or relations in generated graphs, employing advanced methods or tools for synonym detection could improve assessment accuracy. Furthermore, leveraging LLMs for data augmentation in the T2KG construction task shows promise, as our experiments suggest that LLMs can maintain consistency in generating results and propose relevant triples.

Expanding evaluations to a broader range of domains and datasets can provide deeper insights into how various types of data influence model behavior and performance. Combining automated metrics with human evaluation could also offer a richer understanding of model quality, with domain experts providing valuable assessments of the relevance and accuracy of generated graphs. Exploring these directions will contribute to advancing the field of T2KG construction and enhancing the capabilities of language models in producing accurate and contextually appropriate knowledge graphs.

We suggest several future directions for improving the robustness of LLM-based T2KG construction. One key avenue is to explore chain-of-thought (CoT) prompting approaches, which could improve accuracy and reduce hallucinations in multi-step reasoning tasks. Furthermore, an investigation into entity harmonization strategies could lead to more consistent and accurate knowledge graph structures. We also plan to conduct further experiments with newer LLM versions and investigate the impact of hyperparameter tuning on fine-tuning and generalization performance.

Finally, while this study benchmarks various LLM-based approaches for knowledge graph construction, a comparative analysis with traditional NLP-based techniques remains an important area of investigation. Evaluating LLM-based methods alongside classical rule-based or statistical approaches could provide deeper insights into their relative advantages. We leave this exploration as a promising direction for future research.

## Data Availability

Publicly available datasets were analyzed in this study. This data can be found here: https://github.com/Jiuzhouh/PiVe/tree/main/datasets/webnlg20.

## Footnotes

^2^NetworkX - optimal edit paths: https://networkx.org/documentation/stable/index.html.

^3^Hugging Face: https://huggingface.co/.
