# Supplementary material for: Fine-tuning or prompting on LLMs: evaluating knowledge graph construction task
Source: Front Big Data. 2025 Jun 25;8:1505877. doi: 10.3389/fdata.2025.1505877 (PMC12237976; doi:10.3389/fdata.2025.1505877)
Supplement: Supplementary file 1 [file Data_Sheet_1.pdf]

Appendix

June 15, 2025

1 Experimental Setup

Our experiments were conducted using **Google Cloud** with **NVIDIA A100 GPUs** (40GB memory). Due to limited access to cloud resources after the initial experimentation phase, additional fine-tuning and large-scale test cases could not be performed.

For fine-tuning, we employed the QLoRA technique, configuring the hyperparameters as follows: a batch size of **2**, learning rate of **2.5e-5**, and LoRA rank of **8**. The models were fine-tuned on the WebNLG+2020 dataset, and their generalization was evaluated on the KELM-sub dataset.

Future work will explore variations in these hyperparameters to better understand their impact on generalization and performance in the T2KG construction task.

2 Evaluation Metrics Formulations

We mathematically represent the used metrics as follows:

Graph Matching (G-$F1$) Let Mch be the number of matches between predicted and gold graphs. And let $ToGraphs$ be the total number of predicted graphs. Then, the accuracy for entire graph matches Acc_{graph} can be calculated as:

$$ACC_{graph} = \frac{Mch}{ToGraphs}$$

Triples Matcning (T-$F1$) The $F1$ score for triple matches T-$F1$ is calculated in the following:

$$T\text{-}F1 = \frac{2 \times TP}{2 \times TP + FP + FN}$$

Where

- TP is the number of true positive triple matches.
- FP is the number of false positive triple matches.
- FN is the number of false negative triple matches.

Graph Edit Distance (GED) The following equation calculate GED between two given graphs :

$$GED(g1, g2) = \min_{e_1,\ldots,e_k \in \gamma(g1,g2)} \sum_{i=1}^{k} c(e_i)$$

Where:

- GED(g_1, g_2): This represents the graph edit distance between two graphs g_1 and g_2.
- $\min_{e_1,\ldots,e_k \in \gamma(g_1,g_2)}$: This part denotes taking the minimum over all possible edit paths $e_1, \ldots, e_k$ in the set $\gamma(g_1, g_2)$. The set $\gamma(g_1, g_2)$ contains all possible edit paths that transform g_1 into g_2.
- $\sum_{i=1}^{k} c(e_i)$: This part calculates the sum of the costs of each individual edit operation e_i in the selected edit path. The cost function $c(e_i)$ measures the cost or strength of each edit operation. The objective is to find the edit path with the minimum total cost, which represents the least amount of transformation required to convert g_1 into g_2.

In our experiments, we calculate the **overall** GED which is computed as follows:

$$\text{overall\_ged} = \frac{1}{N} \sum_{i=1}^{N} \text{GED}_{\text{ED}_i}$$

Where:

- N is the total number of graphs.
- GED_{ED_i} is the graph edit distance for the ith graph.

Graph BERTScore (G-BS) G-BS takes graphs as a set of edges and solve a matching problem which finds the best alignment between the edges in predicted graph and those in ground-truth graph. Each edge is considered as a sentence and BERTScore is used to calculate the score between a pair of predicted and ground-truth edges, Based on the best alignment and the overall matching score, the computed F1 score is used as the final G-BERTScore. Considering x_i as reference token (entity or relation) and $\hat{x}_i$ as generated token (entity or relation), the complete score matches each token in x to a generated token in $\hat{x}$ to compute recall, and each token in $\hat{x}$ to a token in x to compute precision. A greedy matching is used to maximize the matching similarity score, where each token is matched to the most similar token in the other graph. Then precision and recall are combined to compute an F1 measure. For a reference x and candidate $\hat{x}$, the recall, precision, and F1 scores are:

$$R_{\text{BERT}} = \frac{1}{|x|} \sum_{x_i \in x} \max_{\hat{x}_j \in \hat{x}} x_i^T \hat{x}_j,$$

$$P_{\text{BERT}} = \frac{1}{|\hat{x}|} \sum_{\hat{x}_j \in \hat{x}} \max_{x_i \in x} x_i^T \hat{x}_j,$$

$$F1_{\text{BERT}} = \frac{2 \cdot P_{\text{BERT}} \cdot R_{\text{BERT}}}{P_{\text{BERT}} + R_{\text{BERT}}}.$$

In this work, we use G-BS to compare generated graphs with ground-truth graphs, defining graph matching with a similarity threshold of 95% to introduce GM-GBS (Graph Matching using Graph BERTScore). This approach acknowledges that entities or relations in the generated graph may be synonymous with those in the ground truth graph. A qualitative experinentation using some examples of generated graphs (Figure **??**), shows that even with 95% BERTScore similarity, the generated graph is nearly identical to the ground truth.

To calculate GM-GBS, we follow these steps: Given an array of F1 scores of G-BS $f_1, f_2, \ldots, f_n$ in F1s-BS, the fraction of F1 scores greater than 0.95 is calculated as follows:

1. Let $ToGrs$ be the total number of generated graphs.
2. Let f_m be the count of F1 scores that are greater than 0.95:

$$f_m = \sum_{i=1}^{N} 1(f_i > 0.95)$$

where $1(\cdot)$ is the indicator function, which is 1 if the condition inside is true and 0 otherwise.

3. The fraction of F1 scores greater than 0.95 is given by:

$$GM - GBS = \frac{f_m}{N}$$

Bleu-F1 Score ($F1_{Bleu}$) Let C_{gen} be the count of 4-grams in the generated graph ,
Let C_{ref} be the count of 4-grams in the reference graph, and Let C_{match} be the count of matching 4-grams in

$$P_{Bleu} = \frac{C_{match}}{C_{gen}}$$

$$R_{Bleu} = \frac{C_{match}}{C_{ref}}$$

$$F1_{Bleu} = \frac{2 \times P_{Bleu} \times R_{Bleu}}{P_{Bleu} + R_{Bleu}}$$

ROUGE-F1 Score ($F1_{ROUGE}$) In our experiments, we calculate F1-score for Rouge-2 (bigram), which is presented in the following equation:

$$P_{ROUGE} = \frac{bigram_{cand.} \cap bigram_{ref.}}{bigram_{cand.}}$$

$$R_{ROUGE} = \frac{bigram_{cand.} \cap bigram_{ref.}}{bigram_{ref.}}$$

$$F1_{ROUGE} = 2.\frac{R_{ROUGE}.P_{ROUGE}}{R_{ROUGE} + P_{ROUGE}}$$

Hallucination and Omission. As mentioned before, we calculate hallucination and omission using OEP, which is the optimal edit paths between the gold and predicted graphs. Each edit operation (ei) in OEP represents an action required to transform the predicted graph into the gold graph.

- **Hallucination**: An edit operation e_i is considered a hallucination if it involves adding an entity or a relation that is not present in the gold graph but exists in the predicted graph. To calculate the overall hallucination *hall.*, we use the metric represented by the following equation :

  $$Hall. = \frac{hall}{ToGrs}$$

  Where *hall* is the number of graphs with hallucination, and $ToGrs$ in the total number of generated graphs

  In this work, we calculate also the exact percentages of hallucination and omission between a generated graph and a ground truth graph through qualitative evaluation (Figure **??**). Given a list of tuples lst $= [(g_1, p_1), (g_2, p_2), \ldots, (g_n, p_n)]$, where g_i represents a gold edge and p_i represents a predicted edge:

  1. Let h be the number of hallucinations, where a hallucination is defined as $g_i = \text{None}$: $h = \sum_{i=1}^{n} 1(g_i = \text{None})$

  2. The exact hallucination rate is then calculated as: $\textbf{Hall\_Rate} = \frac{h}{n}$

  Where n is the total number of edges, and $1(\cdot)$ is the indicator function, which is 1 if the condition inside is true and 0 otherwise (Same for Omis_rate).

- **Omission**: An edit operation *ei* is considered an omission if it involves deleting an entity or a relation that exists in the gold graph but is missing in the predicted graph. In our work, we do the same as the hallucination, we calculate the overall omission *omis.*, presented by the following equation :

  $$Omis. = omiss/ToGrs$$

  Where *omiss* is the number of graphs with omission.

  To calculate the exact omission rate between two graphs:
  1. Let o be the number of omissions, where an omission is defined as

$p_i = \text{None}$: $o = \sum_{i=1}^{n} 1(p_i = \text{None})$
2. The exact omission rate is then calculated as: $\textbf{Omis\_Rate} = \frac{o}{n}$
